# Case report: Echocardiographic and computed tomographic features of congenital bronchoesophageal artery hypertrophy and fistula in a dog

**DOI:** 10.3389/fvets.2024.1400076

**Published:** 2024-05-22

**Authors:** Yewon Ji, Jinsu Kang, Suyoung Heo, Kichang Lee, Hakyoung Yoon

**Affiliations:** ^1^Department of Veterinary Medical Imaging, College of Veterinary Medicine, Jeonbuk National University, Iksan, Republic of Korea; ^2^Department of Veterinary Surgery, College of Veterinary Medicine, Kyungpook National University, Daegu, Republic of Korea; ^3^Department of Surgery, College of Veterinary Medicine, Jeonbuk National University, Iksan, Republic of Korea; ^4^Biosafety Research Institute and College of Veterinary Medicine, Jeonbuk National University, Iksan, Republic of Korea

**Keywords:** aortopulmonary shunt, aberrant bronchoesophageal artery, esophageal varices, cardiovascular anomaly, canine

## Abstract

**Introduction:**

Studies on aberrant bronchoesophageal arteries are limited. Herein, we report a case of a multi-origin systemic-to-pulmonary shunt with suspected bronchoesophageal artery hypertrophy and fistula in a dog.

**Case report:**

A 4-year-old castrated male beagle weighing 11 kg underwent routine medical screening. Physical examination revealed a right-sided continuous murmur of grades 1–2. Thoracic radiography revealed a mild cardiomegaly. Echocardiography revealed a continuous turbulent shunt flow distal to the right pulmonary artery (RPA) branch from the right parasternal short axis pulmonary artery view. Computed tomography demonstrated systemic-to-pulmonary shunts originating from the descending aorta at the level of T7–8, the right 5th and 6th dorsal intercostal arteries, and the right brachiocephalic trunk, which formed anomalous networks around the trachea and esophagus that anastomosed into a large tortuous vessel at the level of T6–7 and entered the RPA. Surgical ligation of multiple shunting vessels was performed. Postoperative echocardiography and computed tomography showed decreased left ventricular volume overload and markedly decreased size of the varices. Additionally, most of the shunting vessels were without residual shunt flow.

**Conclusion:**

The present study provides information regarding imaging features and the successful surgical management of multiple systemic-to-pulmonary shunts originating from the descending aorta, right brachiocephalic trunk, and intercostal arteries and terminating at the RPA. Multimodal imaging features after surgical ligation have also been described.

## 1 Introduction

Various cardiovascular malformations associated with systemic-to-pulmonary (L-to-R) shunts have been reported in the veterinary literature. L-to-R shunts can progress to heart failure due to volume overload. The clinical significance of L-to-R shunts depends on their size and the blood flowing through them (1). The types of L-to-R shunts include patent ductus arteriosus (PDA) (2, 3), aortopulmonary window (APW) (4–7), L-to-R arteriovenous fistulae (8), arteriovenous shunts (9), and various atypical L-to-R shunts (10–17). Bronchoesophageal artery (BEA) hypertrophy, also referred to as an aberrant BEA, is a relatively rare type of L-to-R shunt in dogs (10, 11, 14, 15, 17). The BEA usually arises from the fifth right dorsal intercostal artery and courses next to the esophagus, which is known to play a major role in supplying oxygen to the airways and supporting pulmonary structures (18). Normal bronchial arteries from the BEA communicate with the pulmonary artery only through a capillary bed, resulting in a physiologically small amount of L-to-R shunting flow that is hemodynamically insignificant (19). Although aberrant BEAs are known to be abnormal bronchial-to-pulmonary artery communications, the underlying etiology is still not fully understood; however, these anomalies of BEA can result in a significant L-to-R shunt, which can present as similar features on physical and radiographic examinations. Therefore, advanced imaging examinations, such as echocardiography and computed tomography (CT), are essential in distinguishing BEA anomalies from other L-to-R shunts.

To date, five studies on aberrant BEA, BEA hypertrophy, and anomalous BEA have been reported in dogs (10, 11, 14, 15, 17); additionally, there is a lack of reports on BEA anomalies in other animals. However, these cases have differences in the locations of shunt insertion, and there are no reports of cases with right-sided murmurs. In these reports, the echocardiographic and CT characteristics of anomalous BEA have been described; however, to our knowledge, no study in the veterinary literature has described the related postoperative CT features.

Herein, we report a case of a multi-origin systemic-to-pulmonary shunt with suspected BEA hypertrophy and fistula. This report aimed to describe in detail the echocardiographic and CT features of the multiple shunts and changes in postoperative echocardiographic and CT findings.

## 2 Case description

A 4-year-old castrated male beagle weighing 11 kg underwent routine medical screening. The patient had no history of diseases other than chronic otitis externa and no clinical signs indicative of cardiovascular diseases. On physical examination, no abnormalities were observed (temperature, 38°C; heart rate, 110 beats/min; and systolic blood pressure, 150 mmHg); however, a mild right-sided basal continuous murmur of grades 1–2 was noted. Laboratory examinations revealed mildly elevated alanine aminotransferase levels. Thoracic radiography (HF-525 PLUS, ECORAY, Seoul, Korea; kVP 70, mA 200, exposure time 0.03 s) was performed, which revealed an increased vertebral heart score of 11.7 (reference range, 8.7–10.7) and a sternal contact of 3.5 (reference range, 2.5–3.0) on the right lateral view. Generalized cardiomegaly and a slight bulge at the 1–3 o’clock of the cardiac silhouette were identified in the ventrodorsal view. The diameters of the caudal vena cava and pulmonary vessels were within normal ranges.

**Figure 1:**
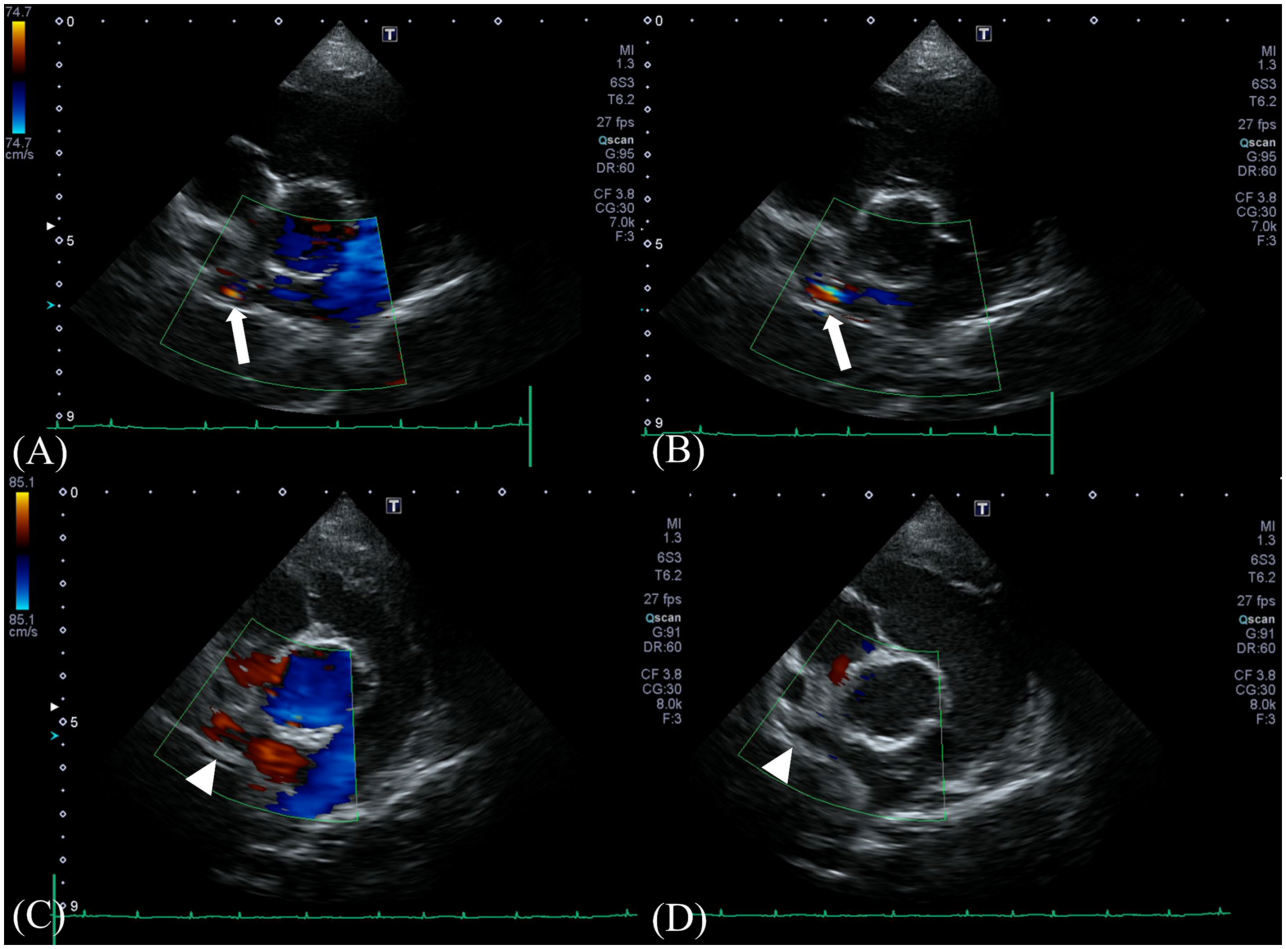
Preoperative Doppler echocardiography at the shunt insertion. Color-flow Doppler examination from a right parasternal short-axis view **(A,B)** reveals a turbulent positive continuous shunt flow entering the distal right pulmonary artery (white arrow) (systolic, 4.18 m/s; diastolic, 2.56 m/s). Postoperative Doppler echocardiography at the preoperative location of the shunt. No residual flow was seen at the location (white arrowhead) **(C,D)**.

Two-dimensional transthoracic echocardiographic examination was performed using a 5-MHz 72 phased array transducer (Aplio 300; Canon Medical System, Europe B.V., Zoetermeer, 73 Netherlands), which revealed the following measurements: left ventricular end-diastolic internal diameter corrected for body weight (LVIDDn), 1.74 (upper limit; <1.7) (20); left ventricular end-systolic internal diameter corrected for body weight (LVIDSn), 1.14 (reference range, 0.74–1.33) (21); end-diastolic volume index (EDVI), 103.11 (upper limit;>100) (22); end-systolic volume index (ESVI), 40.87 (lower limit;<30) (22). These measurements were calculated using the Teichholz method. Additionally, the following were noted: E peak velocity, 91.3 cm/s, 58-117cm/s. (23); E/E’, 10.49 (reference range, 7.3–9.5) (24); and LA:Ao, 0.97 (upper limit <1.6) (20). Collectively, these observations suggested mild left ventricular eccentric hypertrophy. No significant mitral, tricuspid, aortic, or pulmonic regurgitations were observed. From the right parasternal short axis pulmonary artery view, a continuous L-to-R turbulent shunt flow distal to the right pulmonary artery (RPA) showing a mosaic pattern was identified on color Doppler, with a peak velocity of 4.18 m/s and a pressure gradient of 69.89 mmHg on continuous wave Doppler (Figures 1A,B). However, the orifice of the shunt at the typical location of the PDA or the orientation of the shunt could not be identified on echocardiography.

CT (Alexion, TSX-034A, Toshiba Medical Systems, Tochigi, Japan) was performed to further assess the orientation and anatomical features of the suspected shunting vessel. The CT scan was performed under general anesthesia induced using butorphanol 0.2 mg/kg (Butophan; Myungmoon Pharm Co., Ltd., Seoul, Korea) and propofol 6 mg/kg (MCT/LCT 1%, Freefol-MCT; Daewon Pharm Co., Ltd., Seoul, Korea) and maintained with 1.5% isoflurane (Isoflurane; Hana Pharm Co., Ltd., Hwaseong, Korea). The CT parameters were as follows: helical scan mode, 100 kVp, 150 mA, 256 × 256 matrix, rotation time of 0.75 s, and slice thickness of 1 mm. Pre- and post-contrast CT images were acquired. For the contrast medium, 900 mg iodine/kg of iohexol in a total volume of 28 mL (Omnipaque, GE Healthcare, United States) was injected intravenously at a rate of 3 mL/s using a power injector. Post-contrast CT scans were performed when the ascending aorta started to show contrast enhancement and 120 s after the first injection.

**Figure 2:**
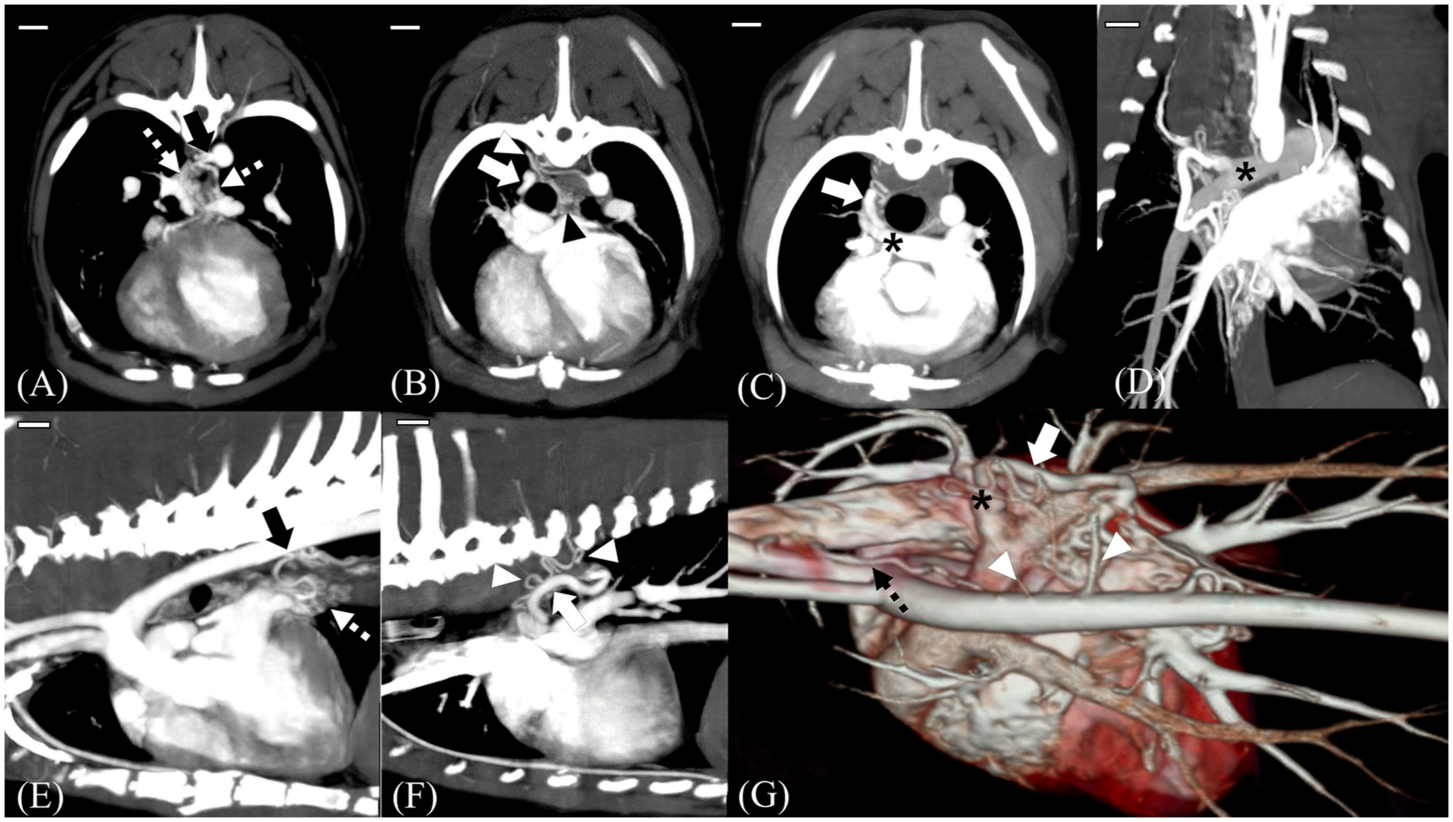
MIP images and a 3D reconstructed image of preoperative computed tomography scans. Scale bars equal 10 mm. Transverse 5-mm-thick slab MIP **(A–C)**, dorsal 10-mm-thick slab MIP **(D)**, sagittal 7.5-mm-thick slab MIP **(E,F)**, and 3D reconstructed images of the heart and adjacent vessels **(G)**. Shunt (black arrow) from the descending aorta at T8 level, which was considered the enlarged left bronchoesophageal artery (BEA) **(A,E)**. Note that in **(E)**, the shunt is supplying the periesophageal network (white dotted arrow). Tortuous vessels (white arrowhead) arising from the fifth and sixth dorsal intercostal arteries course forward and connect to the peritracheal network (black arrowhead) **(B,F,G)**. On dorsal and transverse MIP images, the main shunt (white arrow) entering the distal right pulmonary artery (RPA) (black asterisk) is seen **(C,D)**. 3D reconstructed image showing multiple tortuous vessels (white arrowhead), a network, and the main shunt vessel (white arrow) connected to the distal RPA (black asterisk). Note the aberrant tortuous vessel from the brachiocephalic trunk, which was considered the aberrant right BEA (black dotted arrow) **(G)**.

CT tomography revealed multiple L-to-R shunts (Figure 2). The first shunt (shunt 1) identified was a tortuous vessel originating from the brachiocephalic trunk that ran caudally and anastomosed with the peritracheal network (Figures 2E,G). The second shunt (shunt 2) was caudal to the first one, originated from the thoracic descending aorta at the T8 level, and formed tortuous vessels that eventually connected to the periesophageal network (Figures 2A,B,F). Regarding the third shunt (shunt 3), the vessels arising from the right fifth and sixth dorsal intercostal arteries joined and formed a tortuous shunting vessel that ran cranially and connected to the peritracheal network (Figures 2B–D,F,G). These three shunts were connected to either the peritracheal or periesophageal dense network and eventually anastomosed to a large tortuous vessel connected to the RPA. The measured size of the orifice was 4.1 mm at insertion (Figures 2D,G).

**Figure 3:**
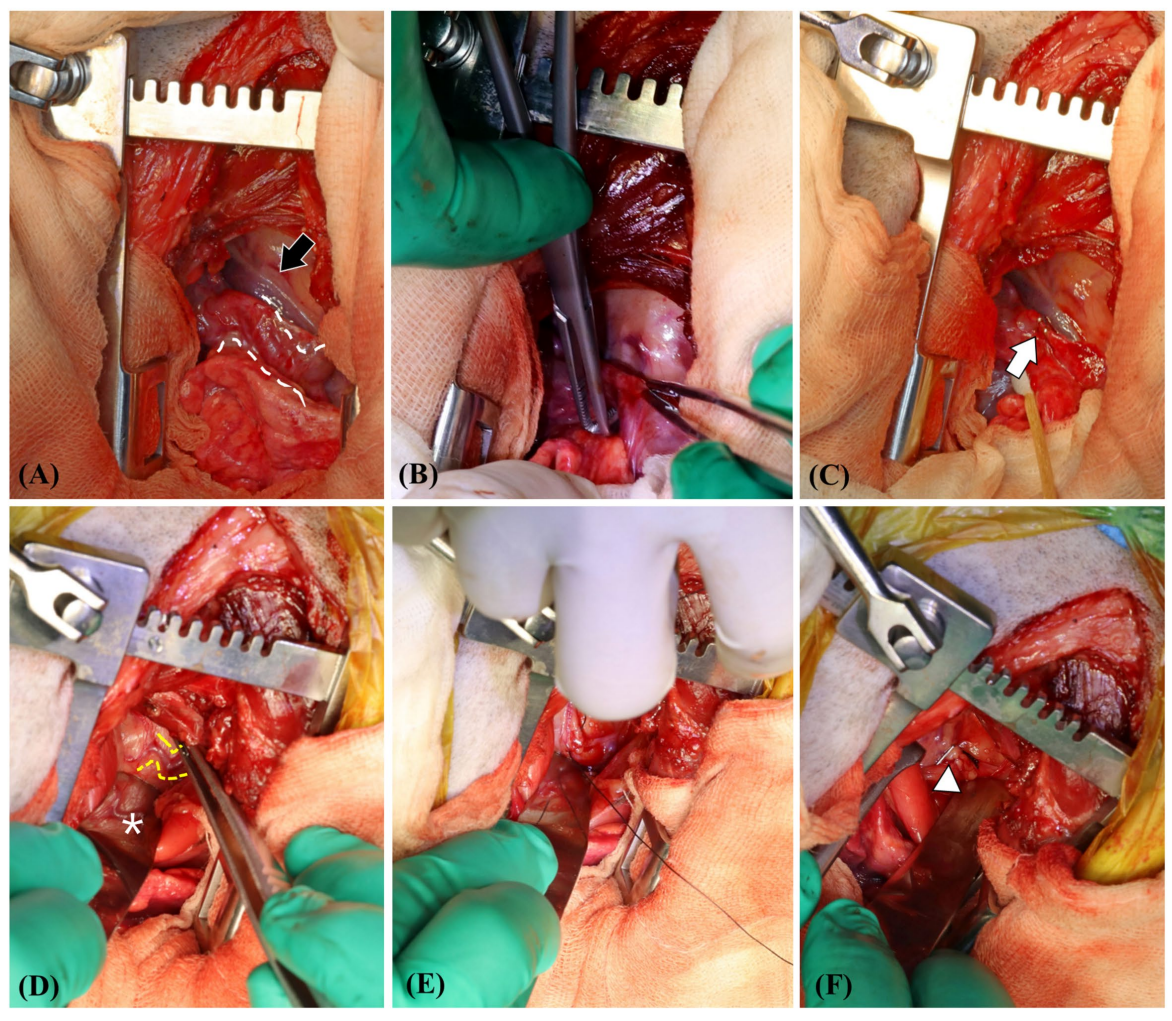
View at the time of surgical ligation of the shunting vessels. The main shunt entering the right pulmonary artery (white dotted line) is identified **(A)**. The caudal vena cava is indicated by the black arrow. Blunt separation and ligation of the shunt are performed using a surgical clip (white arrow) and 5–0 polypropylene **(B,C)**. Identification and ligation of the shunting vessel (yellow dotted line) from the brachiocephalic trunk—which was considered the aberrant form of the right bronchoesophageal artery and right fifth–sixth dorsal intercostal arteries—is performed, and a surgical clip (white arrowhead) is observed. The white asterisk indicates the reflection of the esophagus on the malleable retractor **(D–F)**.

After imaging, the shunts were surgically ligated. Midazolam 0.2 mg/kg (Midazolam, Bukwang Pharm Co., Seoul, Korea) and butorphanol 0.2 mg/kg (Butophan, Myungmoon Pharm Co., Seoul, Korea) were administered intravenously. Intravenous cefazolin 25 mg/kg (cefazolin sodium, Korus Pharm Co., Chuncheon, Korea) was administered as premedication, and general anesthesia was induced with intravenous propofol 6 mg/kg (Provive 1%; Myungmoon Pharm Co., Seoul, Korea) and maintained with sevoflurane (Sevofran, Hana Pharm Co., Seoul, Korea). The dog was positioned in left lateral recumbency, and the right thorax was prepared for aseptic surgery. An intercostal thoracotomy was performed at the 5th right intercostal space, and a shunt entering the right pulmonary artery branch was identified (Figure 3A). The shunt was bluntly separated and ligated using a surgical clip (Hemoclip, Teleflex, Pennsylvania, USA) and 5-0 polypropylene (PROLENE, Ethicon, New Jersey, USA) (Figures 3B,C). Next, the shunts connected to the brachiocephalic trunk and the right 5th-6th dorsal intercostal arteries supplying the periesophageal network were identified, and ligation was performed (Figures 3D–F). Sutures were performed using a routine method, and the dog recovered from anesthesia without any complications. For postoperative analgesia, butorphanol–lidocaine–ketamine was administered (butorphanol at a dosage of 0.02 mg/kg/h, lidocaine [lidocaine hydrochloride, Jeil Pharm Co., Daegu, Korea] at a dosage of 1.5 mg/kg/h, and ketamine [ketamine hydrochloride, Yuhan Corp., Seoul, Korea] at a dosage of 0.6 mg/kg/h) via constant rate infusion.

**Figure 4:**
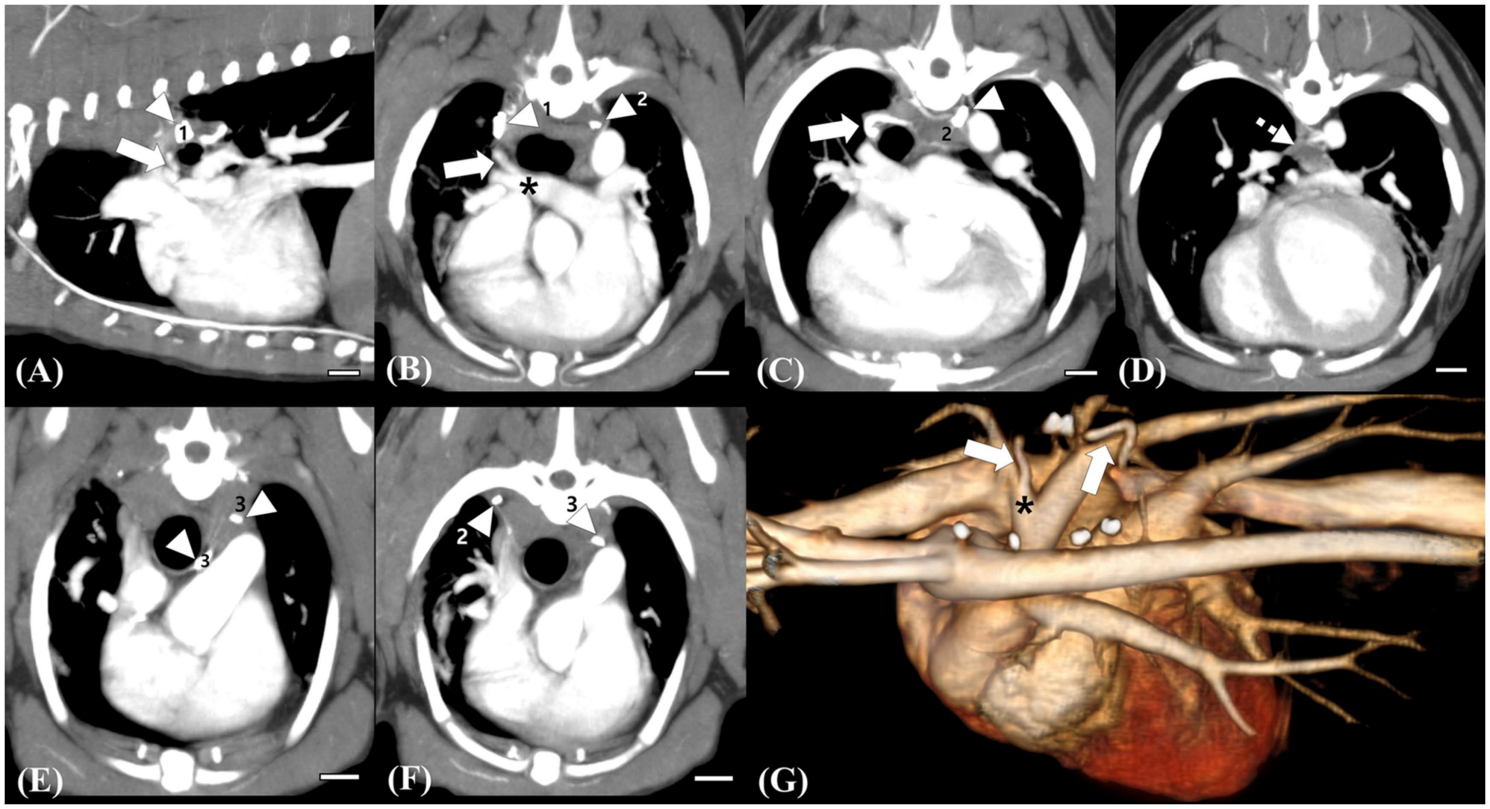
Follow-up computed tomography (CT) images at 12 weeks postoperatively **(A–F)** and a reconstructed 3D image **(G)** for comparison. Scale bars equal 10 mm. The remaining trunk of the ligated vessel (white arrow) connected to the right pulmonary artery, which is supplied by the peritracheal network (white dotted arrow), is identified, but no connection cranial to the surgical clip (white arrowhead, 1) is observed **(A,B,C,G)**. Note the remarkable decrease in the flow of the peritracheal network (white dotted arrow) and the ligated vessel compared with those observed in preoperative CT **(D,G)**. Surgical clip for the ligation of the small tortuous vessel originating from the fifth and sixth intercostal arteries, which were supplying the peritracheal network (white arrowhead, 2) **(C)**. Ligation of the shunting vessel from the brachiocephalic trunk, which was considered the aberrant right bronchoesophageal artery (BEA) supplying the peritracheal network. No remaining flow after the ligation is observed. Surgical clips for the ligation of the suspected aberrant right BEA (white arrowhead, 3) and of the small shunting vessel originating from the fifth dorsal intercostal artery (white arrow, 2) **(C–F)**.

Follow-up echocardiography and CT were performed at 3 weeks and 12 weeks postoperatively. On postoperative echocardiography at 3 weeks and 12 weeks after surgical ligation, no residual shunting of the right pulmonary artery was observed (Figures 1C,D). Additionally, echocardiographic parameters showed a diminution of the left ventricular volume overload (preoperative LVIDDn 1.74 to postoperative LVIDDn 1.46; preoperative EDVI 103.11 to postoperative EDVI 67.41). Moreover, decreases in E peak velocity from 91.3 cm/s to 44.70 cm/s, and E/E’ from 10.49 to 6.67 cm/s were observed, which indicated a decrease in left atrial pressure. CT at 3 weeks postoperatively showed no residual enhancement caudal to the ligation site of shunt 1 originating from the brachiocephalic trunk or shunt 2 originating from the descending aorta. Shunt 3 originating from the fifth and sixth right dorsal intercostal arteries, showed no cranial or caudal residual flow to the clip at the two ligation sites; however, mild residual enhancement of the vessel connected to the peritracheal network was observed. The shunting vessel anastomosed from the three shunts and the peritracheal–periesophageal network were observed; however, no connection between the aorta and the RPA was identified (Figure 4). No remarkable changes or aneurysms were observed on the follow-up CT at 12 weeks postoperatively.

## 3 Discussion

An L-to-R shunt can result in left ventricular volume overload, and treatment options vary according to shunt type; thus, a definitive diagnosis of the shunt is important. To date, several anomalous aortopulmonary shunts that differ from typical PDA or APW, which comprise the majority of L-to-R shunts in dogs, have been reported in the veterinary literature (10–16). Herein, the physical, radiographic, echocardiographic, and CT characteristics of BEA hypertrophy compared to other L-to-R shunts are discussed.

Few reports have described the features of BEA (25, 26) and anomalous L-to-R communication associated with aberrant BEA in dogs (9–11, 14, 15). Previous reports have demonstrated shunts between the BEA and the pulmonary artery or branches suspected to be aberrant branches of the BEA connected to the main pulmonary artery (MPA) (10, 11, 15). In one study, all cases of aberrant BEA coexisted with a network of closely related tortuous vessels. Additionally, it described the features and patterns of congenital and acquired BEA hypertrophy and concurrent L-to-R artery communications (14). A recent study described the imaging features of anomalous bronchial and non-bronchial arterial blood supply to the lungs in dogs and proposed a classification of BEA hypertrophy. The imaging features of congenital BEA hypertrophy include an enlarged left BEA from an enlarged intercostal artery between T5 and T8; an aberrant origin of the right BEA from the right brachiocephalic trunk; the presence of a large and tortuous vessel between the dense peritracheal–periesophageal network and the MPA through an orifice; and no evidence of lung or pleural diseases (14).

Herein, one physical characteristic was a mild continuous murmur heard at the basal level of the right heart. Continuous murmurs are usually heard in PDA and APW (2, 4), generally at the base and apex (4, 27). All previous cases of BEA hypertrophy had various grades of left-sided murmur, either apical or basal (11, 14, 15, 17); however, no study has reported BEA hypertrophy with a right basal murmur. In previous reports regarding BEA hypertrophy, shunt insertion locations were the proximal left pulmonary artery, between the aorta and the pulmonary artery, and the proximal RPA. Herein, the location of the main shunt entering the RPA was distal rather than proximal; this may be a reason for the different murmur locations. These findings indicate that BEA hypertrophy can have various shunt insertion locations, and murmur locations can vary according to the location. In addition to relatively common congenital anomalies with right-sided murmurs, such as ventricular septal defects (VSDs) (28), rare cardiovascular anomalies such as the aorta-right atrial tunnel (29) can present with a right-sided continuous murmur. Therefore, the differential diagnosis, in the case of a continuous right-sided murmur, should include BEA hypertrophy, aorto-right atrial tunnel, and VSD. However, murmur alone is insufficient to diagnose BEA hypertrophy; echocardiography and CT should be performed for a definite diagnosis.

Herein, distinct echocardiography and CT features enabled the differentiation of the condition from other cardiovascular anomalies. Because the location of the main shunt entering the distal RPA was different from the level of the ligamentum arteriosum, it could be distinguished from PDA. Tortuous communicating vessels were also identified. Because APW is characterized by an opening or window without tortuous vessels or significant length of communication (30), it could be differentiated from APW. This case also showed three tortuous vessels originating from different locations entering the dense peritracheal and periesophageal networks, which eventually formed a large tortuous vessel that entered the RPA. One of the tortuous vessels was connected to the suspected aberrant BEA originating from the fifth and sixth intercostal arteries and entered the peritracheal network. Our findings were similar to those of a previous report on congenital BEA hypertrophy; therefore, the present case was considered to have congenital BEA hypertrophy.

However, herein, as the main shunt entered distal to the RPA, there were some differences compared with previous reports. In a review of BEA hypertrophy and aberrant BEA, only two dogs had shunts entering the RPA, while others had shunts entering the left pulmonary artery, usually at the proximal part or between the aorta and the pulmonary artery caudal to the ligamentum arteriosum (11, 14, 15). In both cases, shunts entering the RPA were proximal, not distal (14, 15).

Shunt insertion location can be critical when L-to-R shunt identification is based solely on echocardiography, especially when the patient is asymptomatic. Because the efficacy and accuracy of the diagnosis from echocardiography are affected by the experience of the clinician performing echocardiography (31), uncommon shunts with atypical origins may be neglected and remain unidentified. It is relatively easier to identify shunts entering the MPA or proximal branches on the echocardiography on the basic right parasternal short-axis view at the level of the MPA, but they can be missed when shunts with a small amount of flow enter the distal branches. Therefore, it is important to check for anomalous shunt flows not only at the location of typical shunts but also distal to them from modified views, if needed.

Herein, subsidiary anomalies were observed compared to typical BEA hypertrophy alone. On CT examination, a slight difference was identified cranially, where the enlarged left BEA arose. The fifth and sixth dorsal intercostal arteries did not course directly but tortuously from the aorta, from which two tortuous arteries arose. These two tortuous vessels coursed and eventually connected as a tortuous vessel and entered the peritracheal network. Despite the unknown etiopathology of BEA hypertrophy and concurrent peritracheal–periesophageal network, the left BEA and fifth, sixth, and eighth dorsal intercostal arteries develop as branches of the dorsal intersegmental arteries or their ventral branches during the development of the vascular system, and the suspected BEA hypertrophy and additional anomalies could be the result of secondary connections during the embryonal stage (18).

On follow-up echocardiography at 3 weeks and 12 weeks postoperatively, no residual flow and decreased LVIDDn, EDVI, E peak velocity, E/A ratio, and FS were observed. Decreased LVIDDn and EDVI were considered to result from the decreased left ventricular volume overload. Decreased left ventricular preload and filling pressure after surgical ligation could result in a decreased E peak and E/A ratio (32). Additionally, decreased FS might have resulted from decreased diastolic volume without a significant change in systolic volume and was considered a temporary postoperative status (32).

Although surgical ligation of suspected BEA hypertrophy and fistulas has been reported in two case reports (10, 15), there is a lack of studies describing changes in CT findings after surgical ligation of the shunts. Herein, follow-up CT scans at 3 weeks and 12 weeks after surgical ligation showed no residual flow of the large shunting vessel entering the RPA and no residual flow caudal to the ligation site of shunt 1, which was considered an anomalous origin of the right BEA connecting and supplying the peritracheal network. Shunt 3, which supplied the peritracheal network, was also ligated; the amount of the peritracheal network was markedly decreased, but a small amount of flow was observed. Shunt 2, considered an aberrant form of a hypertrophied left BEA, was not perfectly ligated, which was suspected to be the reason for the mild residual flow of the periesophageal network.

A previous report described cases that underwent echocardiography, angiography, and CT examinations before the surgical ligation of shunts (15). In three of the four dogs, only the large main shunts entering the pulmonary artery were surgically ligated, which resulted in immediate shrinkage of some or all tortuous vessels. One dog had at least two vessels in addition to the major vessel of aortic origin that underwent separate ligation, similar to the present case. However, the origins and characteristics of these additional vessels were not fully elucidated, and no postoperative CT was performed. Herein, a small amount of residual flow was observed in the tortuous network postoperatively; however, no residual flow was identified on echocardiography. As residual flow can only be identified on CT and not on echocardiography, and tortuous networks cannot be assessed on echocardiography, follow-up CT examination, in addition to echocardiography, is needed to thoroughly assess residual flow and the existence of remaining shunts.

This study has some limitations. First, on the follow-up CT, a mild residual flow in the periesophageal network was observed, which could have led to an aneurysm. However, no aneurysm was identified at the 3-month follow-up. In addition, it has been reported that the resolution of transient deterioration of left ventricular systolic function could be seen after 6 months of surgical or interventional ligation of L-to-R shunts (32). Second, no follow-up echocardiographic examination after 12 weeks was performed in this case. However, as in the present case, although postoperative echocardiographic parameters indicated resolution of the left ventricular volume overload, there was an insignificant amount of residual flow from the tortuous network. Even if a small amount of residual flow remains in the peritracheal–periesophageal network, the surgical ligation of major shunt vessels can clinically help in the resolution of LV volume overload.

## 4 Conclusion

This report describes the preoperative and postoperative physical, echocardiographic, and CT features of BEA hypertrophy. BEA hypertrophy should be included as a differential diagnosis when a right-sided murmur is noted, a mosaic pattern at the distal RPA is observed on echocardiography, and a shunt of systemic arterial blood flow inserted into the pulmonary artery is seen on CT examination. Surgical ligation is a practical method for the occlusion of L-to-R shunts in BEA hypertrophy, which can decrease the volume overload of the heart and result in a remarkable decrease in the number of peritracheal and periesophageal networks.

## Data availability statement

The raw data supporting the conclusions of this article will be made available by the authors, without undue reservation.

## Ethics statement

Ethical approval was not required for the studies involving animals in accordance with the local legislation and institutional requirements because the manuscript is a case report of an imaging diagnosis and surgery of a client-owned companion animal, written informed agreement was obtained from the client instead. Written informed consent was obtained from the owners for the participation of their animals in this study.

## Author contributions

YJ: Conceptualization, Data curation, Formal analysis, Funding acquisition, Investigation, Methodology, Project administration, Resources, Software, Supervision, Validation, Visualization, Writing – original draft, Writing – review & editing. JK: Conceptualization, Investigation, Methodology, Project administration, Validation, Writing – review & editing. SH: Writing – review & editing. KL: Conceptualization, Supervision, Validation, Writing – review & editing. HY: Conceptualization, Data curation, Formal analysis, Funding acquisition, Investigation, Methodology, Project administration, Resources, Software, Supervision, Validation, Visualization, Writing – original draft, Writing – review & editing.
